# Cardiovascular toxicities associated with chimeric antigen receptor T-cell therapy

**DOI:** 10.3389/fphar.2025.1578157

**Published:** 2025-05-08

**Authors:** Jia-Hui Liu, Kun-Yao Liu, Xiang Zhao, Xin Zhou, Yichuan Jiang

**Affiliations:** ^1^ Cancer Center, The First Hospital of Jilin University, Changchun, China; ^2^ Cancer Research Institute of Jilin University, The First Hospital of Jilin University, Changchun, China; ^3^ International Center of Future Science, Jilin University, Changchun, China; ^4^ Department of Pharmacy, China-Japan Union Hospital of Jilin University, Changchun, China; ^5^ Department of Clinical Pharmacy, The First Hospital of Jilin University, Changchun, China

**Keywords:** CAR T cell, cardio-oncology, cytokine release syndrome, cardiotoxicity, chimeric antigen receptor

## Abstract

Chimeric antigen receptor (CAR) T-cell therapy has emerged as a groundbreaking immunotherapeutic approach, particularly for oncohematological patients who are refractory to conventional treatments. As clinical trials expand the applications of CAR T-cell therapy beyond hematologic malignancies, a critical understanding of its associated toxicities, particularly cardiovascular complications, becomes imperative. This review synthesizes current literature on the interplay between cytokine release syndrome (CRS) and cardiotoxicity related to CAR T-cell therapy, emphasizing the potential severity of these adverse events. While significant progress has been made in managing CRS, the cardiac manifestations—ranging from mild events to life-threatening complications—remain underreported in pivotal studies. We explore the incidence and nature of cardiotoxicity in real-world and clinical trial settings, identify risk factors contributing to cardiovascular events, and propose guidelines for pre-therapy evaluations, post-infusion monitoring, and management strategies. By highlighting the urgent need for heightened awareness and proactive care, this review aims to enhance patient safety and optimize outcomes in the evolving landscape of CAR T-cell therapy.

## Introduction

Cancer has become the leading cause of death globally, which has a profound impact on the health of the population, the national economy, and the progress of society around the world (Hafeez et al., 2020; Huang et al., 2024). Over the past three decades, remarkable progress has been achieved in cancer treatment, evolving from surgery, radiotherapy, and chemotherapy to targeted therapy, and more recently, immunotherapy (Baskar et al., 2012). Immunotherapy encompasses a range of strategies, such as immune checkpoint inhibitors (ICI), Chimeric antigen receptor (CAR) T-cell therapy, bispecific T cell engaging antibodies, cytokine-based treatments (e.g., high-dose interleukin-2 [IL-2] and interferon-α[IFN-α]), and monoclonal antibody therapies (June and Sadelain, 2018). Among these immunotherapies, CAR T-cell therapy represents an innovative immunotherapy approach that has achieved remarkable results in the treatment of hematological malignancies (Huang et al., 2024). Up to now, the CAR T cells approved by the United States Food and Drug Administration (FDA) are designed to target the CD19 antigen, which is highly expressed in B-cell malignancies (Crees and Ghobadi, 2021). However, data from clinical trials and observational studies indicate that this therapy may be associated with a range of toxicities, especially cardiac toxicities (Brudno and Kochenderfer, 2019). In this review, we summarized the adverse cardiac events observed in clinical trials of CAR T-cell therapy. We found that the underlying mechanisms for their occurrence seem to be related to the abnormal inflammatory activation observed in cytokine release syndrome (CRS) (Camilli et al., 2023a). Based on the key pathways involved in the currently understood mechanisms of CRS, we provide monitoring and management strategies. These strategies aim to enhance the safety of CAR T-cell therapy and expand its scope of application (Brudno and Kochenderfer, 2024).

## Chimeric antigen receptor (CAR) T-cell therapy

T lymphocytes, also known as T cells, are a subtype of lymphocytes that play a vital role in the adaptive immune system by defending against disease-causing pathogens as well as protecting the body from abnormal cells (such as tumor cells), which is called immune surveillance (Schmid et al., 2006). Normal T cells trigger an immune response when they recognize antigens presented to them by antigen-presenting cells (APCs) that are coupled to major histocompatibility complex (MHC) molecules. Cancer cells can also express MHC molecules on their surface membranes (Darragh and Karam, 2022). In addition, conventional T cell therapy requires co-stimulation of other immune cells to trigger cytokine release, cytotoxic activity, and proliferation to be sufficient to induce an immune response (Gray et al., 2006).

**FIGURE 1:**
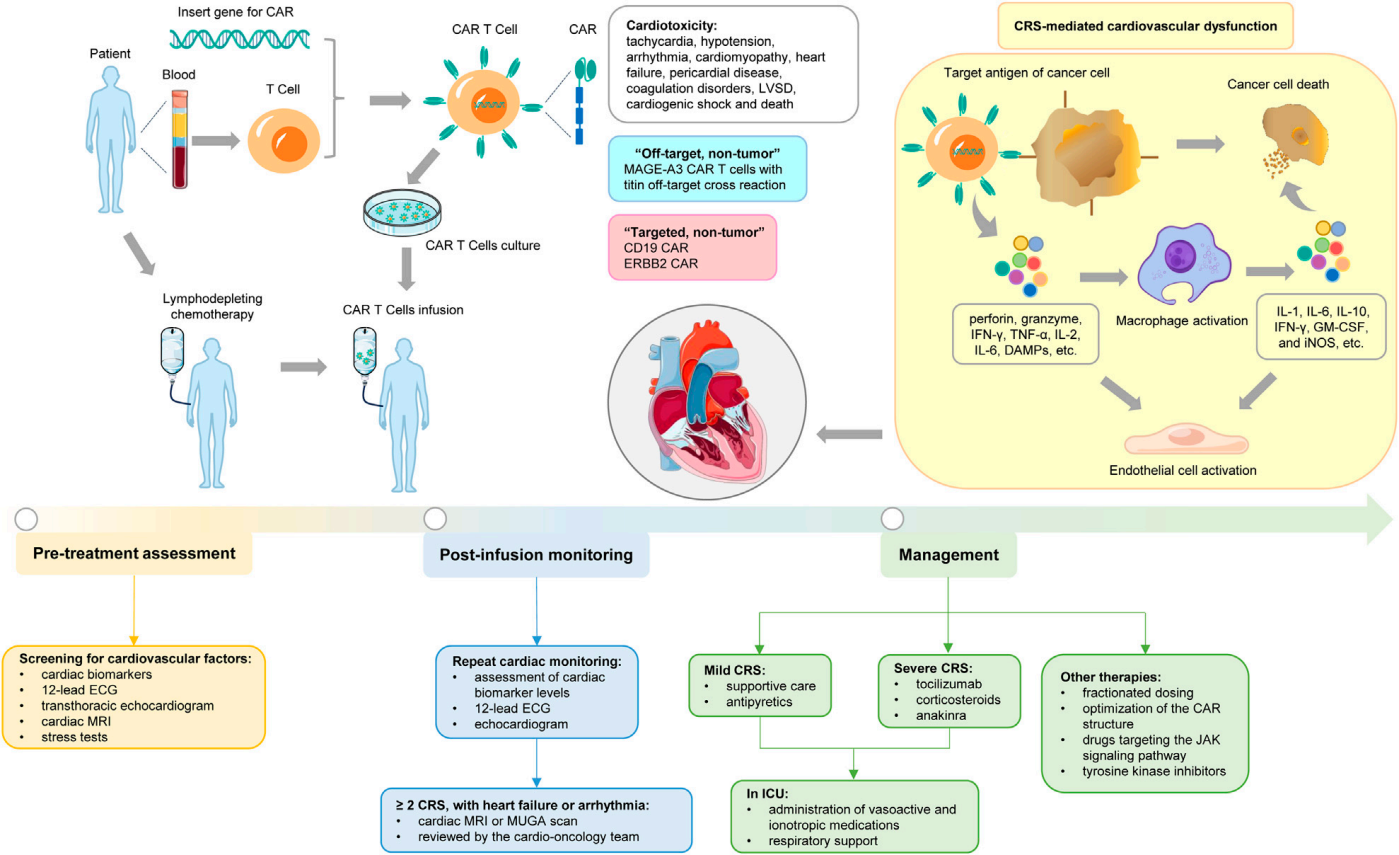
Cardiovascular toxicities associated with CAR T-cell therapy. The figure illustrates the general CAR T-cell therapy and the specific mechanisms of induced cardiotoxicity, as well as the associated assessment, monitoring, and management.

CAR T-cell therapy, an innovative adoptive cell therapy that represents a new paradigm for cancer management, gained attention in 2012 after it was used to treat a pediatric patient with relapsed acute lymphoblastic leukemia (ALL) (Gill, 2023). It stimulates a direct anti-tumor response through the cell therapy mechanism of *in vitro* transgenic T cells, and the preparation process takes approximately 2–4 weeks (Nunes et al., 2024). CAR T cells are not existing products, but autologous T cells are isolated from peripheral blood of patients and collected by leukocyte apheresis (Padmanabhan, 2018). Lentivirus or γ retrovirus vectors are used for gene modification, allowing the required engineered CAR sequence to be inserted and integrated into the genome of autologous T cells, thereby leading to the expression of CAR on the surface of T cells, for example, CD19 (Freyer and Porter, 2020; Perry and Rayat, 2021). The transfected cells are then amplified in the laboratory using various methods that rapidly proliferate *in vitro* to produce therapeutic amounts, such as artificial APCs expressing CD80 and stimulating cytokines such as IL-15 (Yoshikai and Nishimura, 2000; Herrero-Beaumont et al., 2012). After expanding these cells *in vitro*, they are reintroduced to the patient, who receives lymphodepleting conditioning regime (LDC) with cyclophosphamide or fludarabine 3–5 days prior to administration, which inhibits the patient’s regulatory immune cell population to promote the activation and proliferation of the transfused CAR T cells *in vivo* (Shank et al., 2017). Thus, the therapeutic effect of CAR T cells was enhanced. Preoperative administration of acetaminophen and diphenhydramine should be given 30–60 min before CAR T cell infusion (Geiger and Howard, 2007). Shortly after infusion, the CAR T cells bind to the target antigen and are activated, causing them to rapidly multiply in the body and release inflammatory cytokines, which in turn recruit other immune cells to the tumor site, such as macrophages. These CAR T cells also exert cytotoxic effects by releasing cytotoxic particles containing granzyme and perforin, and directly stimulate tumor cell apoptosis by activating Fas/Fas-L and TNF-R pathways that lead to tumor cell destruction (Figure 1) (Hay and Slansky, 2022). Most patients receive CAR T cell transfusions and are monitored in the hospital for several days to weeks (Bachier et al., 2019).

CAR is a synthetic transmembrane protein expressed on the surface of immune effector cells and consists of 4 domains: extracellular tumor antigen recognition domains, a hinge point connecting extracellular components to cytoplasmic elements, transmembrane domains, and intracellular domains derived from T cell receptor (TCR) that trigger signaling mechanisms leading to T cell activation (CD3ζ) or co-stimulatory domains (e.g., CD28, 4-1BB and OX-40) (Maher et al., 2002; Sadelain et al., 2013). Single-chain variable fragments of immunoglobulins are often used to recognize target tumor antigens due to their high binding specificity, subsequently leading to engineered T cell activation independent of the MHC (Dai et al., 2016; Mohanty et al., 2019). In addition to their immediate anti-tumor benefits, CAR T cells also provide long-term benefits as they promote immune surveillance to prevent tumor recurrence by continuously targeting malignant cells and assisting in the activation of tumor-infiltrating lymphocytes (June and Sadelain, 2018). It is important to note that the activity of CAR proteins can vary depending on the overall build design, and predicting the effectiveness of CAR T-cell therapy infusion is difficult because each CAR construct may have a different infusion effect (Letscher and Reddy, 2024). But the ideal target, as well as amplification to achieve sufficient numbers of CAR T, will promote targeting of rapidly proliferating malignancies (Jadlowsky et al., 2025).

In clinical trials, CAR T-cell therapy has shown significant efficacy in the treatment of a variety of highly relapsed/refractory hematological malignancies (Goyco Vera et al., 2024). In 2017, the FDA approved 2 CAR T-cell therapies: tisagenlecleucel and axicabtagene ciloleucel (U.S. Food and Drug Administration, 2017; U.S. Food and Drug Administration, 2017), which are CAR T cells targeting the B-cell receptor CD19, respectively for the treatment of pediatric and young adults with relapsed/refractory B-ALL and diffuse large B-cell lymphoma (DLBCL). Then, in July 2020, the FDA approved the CAR T-cell therapy brexucabtagene autoleucel for the treatment of adult relapsed/refractory mantle cell lymphoma (MCL) (U.S. Food and Drug Administration, 2020). Lisocabtagene maraleucel was approved in February 2021 for the treatment of adult relapsed/refractory large B-cell lymphoma (LBCL) (U.S. Food and Drug Administration, 2021). The first approved non-CD19 cell therapy was ldecabtagene vicleucel, created against B cell maturation antigen (BCMA), which has shown effectiveness in treating multiple myeloma (Ali et al., 2016; Brudno et al., 2018). This treatment fundamentally changes the paradigm of cancer treatment. Data from the World Health Organization’s International Agency for Research on Cancer (IARC) for 2023 show that five-year survival rates for myeloma and non-Hodgkin lymphoma improved from 32% and 56% in 1995–1997 to 58% and 74% in 2012–2018, marking significant clinical achievements (Siegel et al., 2023). As a result of these successes, CAR T cell immunotherapy is receiving increasing attention from scientists, clinicians, the pharmaceutical industry, and the general public, and several new clinical trials have been initiated to expand the clinical applicability of CAR T-cell therapy for cases such as chronic lymphocytic leukemia (CLL) and other solid malignancies, for example hepatocellular carcinoma, breast cancer, or pancreatic cancer (Mohanty et al., 2019). In addition, the success of hematological malignancies has stimulated research on CAR T-cell therapy for the treatment of autoimmune diseases, HIV and other infections, and transplant rejection (Maldini et al., 2018).

## Cardiotoxicity associated with CAR T-cell therapy

While CAR T-cell therapy has shown strong clinical efficacy in relapsed/refractory hematological malignancies, with a response rate of up to 90%, it is limited by the incidence of severe life-threatening cardiotoxicity of up to 26%, as most patients considered for this treatment have cardiovascular comorbidities, and had previously received high doses of cardiotoxic chemotherapy and radiation (Neelapu, 2019). Manifestations may include tachycardia, hypotension, arrhythmia, cardiomyopathy, heart failure, pericardial disease, coagulation disorders, left ventricular systolic dysfunction (LVSD), cardiogenic shock and death, and may occur at various time points after CAR T-cell therapy has begun (Lyon et al., 2022). Up to now, several prospective and retrospective reports have highlighted the association between CAR T-cell therapy and adverse cardiovascular events in children and adults, significantly affecting morbidity and mortality (Burstein et al., 2018; Alvi et al., 2019; Ganatra et al., 2020; Lefebvre et al., 2020).

### In pediatric and young adult patients

In 2018, Burstein et al. reported the first study on cardiotoxicity following CAR T-cell therapy, a retrospective analysis of 98 pediatric and young adult patients receiving tisagenlecleucel for relapsed/refractory ALL. 24 patients developed hypotension requiring vasopressor support, with a mean duration of onset of 4.6 days, and all had grade 3–4 CRS. Of these, 21 patients had life-threatening symptoms requiring tozzizumab, and 10 patients had LVSD revealed by echocardiography. Among these, 7 patients had persistent LVSD at the time of discharge, but during follow-up, the disorder had gradually resolved in 6 patients without any cardiac events leading to death (Burstein et al., 2018).

In July 2020, Shalabi et al. reported a second retrospective study of the effects of CD19 CAR T-cell therapy on cardiac function in 52 pediatric and young adult patients with relapsed/refractory B-ALL or non-Hodgkin lymphoma. All patients were previously exposed to anthracycline chemotherapy. 37 patients developed CRS, 9 had hypotension requiring vasopressor support, and 7 received tozizumab or steroids. Of the patients who developed CRS, 6 had post-infusion echocardiography showing cardiac insufficiency, of which 4 had grade 3–4 CRS. Elevated troponin was observed in 4 of 13 patients with systolic dysfunction, and the left ventricular ejection fraction (LVEF) decreased from 64% to 20%. 4 of the 6 patients with cardiac insufficiency had resolved by day 28 after CAR T cell infusion, and the remaining 2 patients showed complete cardiac recovery at 3 months follow-up. Interestingly, these 2 patients had the highest exposure to anthracyclines prior to treatment (Sadelain et al., 2013; Shalabi et al., 2020).

Thus, clinical data in pediatric and young adults suggest that cardiotoxicity following CAR T-cell therapy, though severe in some cases, is generally reversible and does not have lasting effects.

### In adult patients

In June 2020, Lefebvre et al. reported a retrospective study of the effects of CD19 CAR T-cell therapy on cardiac function in 145 adult patients with DLBCL (30%), ALL (25%), or CLL (45%). In the study, 60% of patients were exposed to anthracyclines. Patients received commercially available (axicabtagene ciloleucel and tisagenlecleucel) or non-commercially available CAR T cells. The researchers identified 41 cases of major cardiac adverse events (MACE) that developed 11 days after CAR T cell infusion, with grade 3–4 CRS having a higher risk. 22 patients had heart failure events, 12 episodes of atrial fibrillation, 2 other arrhythmia events, 2 episodes of acute coronary syndrome, and 2 cardiogenic deaths. In addition, MACE was associated with statins, aspirin, insulin, and patients with prior atrial fibrillation and elevated creatinine at baseline (Lefebvre et al., 2020). Therefore, these results suggest that patients with high-risk cardiovascular characteristics are more likely to develop MACE.

In October 2020, Ganatra et al. published a retrospective review of 187 adult patients treated with CD19 CAR T cells for non-Hodgkin lymphoma to detect the incidence of cardiomyopathy. 97 percent of the patients received axicabtagene ciloleucel. A total of 155 patients had CRS, of which more than 50% had grade ≥2 CRS. Of the 116 patients who underwent continuous echocardiography, 12 developed new or worsening cardiomyopathy, manifested by a mean decrease in LVEF from 58% to 37% at 12.5 days after CAR T cell infusion. In these 12 patients with cardiomyopathy, 6 LVEF recovered to normal, 3 partially recovered, and 3 cardiogenic deaths. Similar to previous studies, most patients with cardiomyopathy had ≥ grade 2 CRS. In addition, the researchers note that older patients with hyperlipidemia, coronary artery disease, and the use of renin-angiotensin inhibitors and β blockers are at increased risk for cardiomyopathy. Interestingly, there was no significant difference in exposure to anthracyclines or radiotherapy between patients with cardiomyopathy and those without (Ganatra et al., 2020).

Thus, clinical data in adults, compared to pediatrics and young adults, suggest that cardiotoxicity in cases of grade 2 or higher CRS is not always reversible, and that patients with increased cardiovascular risk factors are at greater risk of fatal cardiac events.

## CRS-mediated cardiovascular dysfunction

Cardiotoxicity may be due to the direct or indirect effects of the infusion of CAR T cells, and the mechanism revolves around three pathways: “targeted, non-tumor” toxicity, “off-target, non-tumor” toxicity, and CRS-mediated cardiovascular dysfunction (Joseph et al., 2025). In “targeted, non-tumor” toxicity, T cells target normal tissue cells that express tumor-associated antigens, such as B-cell aplastic anemia observed with CD19 CAR, and pulmonary edema caused by ERBB2 CAR (Chen et al., 2022). In “off-target, non-tumor” toxicity, CAR T cells may also attack the heart by cross-reacting with myocardial antigens from normal tissue (Nunes et al., 2024). Linette et al. reported that 2 patients receiving CAR T-cell therapy for melanoma targeting melanoma-associated antigen 3 (MAGE-A3) developed fatal myocarditis and cardiogenic shock. This is because CAR T cells exhibit an off-target cross-reaction to titin, a sarcomere protein expressed in the myocardium, which contains an epitope similar to that of MAGE-A3. Notably, MAGE-A3 is widely expressed in melanoma (Linette et al., 2013).

The key driver of cardiotoxicity associated with CAR T-cell therapy is thought to be primarily CRS resulting from over-activation of the immune system (Asnani, 2018; Zhao et al., 2018). In retrospective analyses of patients receiving CAR T treatment, the severity of CRS is typically closely associated with the occurrence of cardiotoxicity (Lee et al., 2014; Lefebvre et al., 2020). Cardiac events are mainly observed in patients with CRS of grade ≥2 and are usually caused by elevated troponin levels (Ghosh et al., 2020). Potential disease burden, tumor load, the type of CAR T construct, the infused dose of CAR T cells, and the addition of fludarabine in the lymphodepletion regimen are key risk factors for the severity of CRS (Maude et al., 2014).

In 2018, the American Society for Transplantation and Cellular Therapy (ASTCT) released a consensus system for the clinical grading of the severity of CRS, which is mainly based on fever, hypoxia, and hypotension (Table 1). CRS is defined as any hyper-physiological reaction caused by the activation or involvement of endogenous or infused T cells and/or other immune effector cells after any immunotherapy (Lee et al., 2019). Clinically, the symptoms of CRS range from mild to severe and can rapidly progress from fever, weakness, headache, rash, diarrhea, and musculoskeletal pain to hypoxia, hypotension, tachycardia, left ventricular systolic dysfunction, arrhythmia, elevated troponin, coagulation disorders, pericardial disease, decompensated heart failure, cardiogenic shock, and CRS may develop into a life-threatening condition (Davila et al., 2014; Maude et al., 2014; Lee et al., 2015; Ali et al., 2016).

**TABLE 1:** Consensus cytokine release syndrome grading scale-the American society for transplantation and cellular therapy.

Clinical symptoms	Grade1	Grade2	Grade3	Grade4
Fever (Temperature)	≥38°C	≥38°C	≥38°C	≥38°C
with Hypotension	None	Yes, but do not require vasopressors	Yes, and require vasopressors with or without vasopressin	Yes, and require multiple vasopressors excluding vasopressin
and/or Hypoxia	None	Yes, and require low-flow (O_2_ ≤ 6 L/min) nasal cannula	Yes, and require high-flow (O_2_ > 6 L/min) nasal cannula, non-rebreather, or venturi mask	Yes, and require positive pressure (CPAP, BiPAP, intubation, and mechanical ventilation)

BiPAP, biphasic positive airway pressure; CPAP, continuous positive airway pressure.

CRS typically begins within the first week after CAR T cell administration and can persist for up to 10–14 days, although symptoms may be delayed (Santomasso et al., 2019). Clinical data indicate that the incidence of CRS in patients receiving CAR T treatment ranges from 35% to 94%, with the incidence of severe CRS (≥ grade 3) ranging from 10% to 30% (Grigor et al., 2019). In fact, every patient receiving CAR T-cell therapy is expected to, and indeed hopes to, develop some degree of CRS, as grade 1 disease indicates a response to the treatment.

CRS is a systemic disease induced by the over-activation of immune effector cells and the hyper-physiological levels of various pro-inflammatory cytokines (Lee et al., 2019; Cobb and Lee, 2021). It is characterized by a pro-inflammatory cytokine storm following cell infusion and its interaction with the tumor microenvironment (Hughes et al., 2024). After recognizing tumor antigens, CAR T cells are activated and secrete large amounts of perforin, granzyme, and inflammatory cytokines such as IFN-γ, tumor necrosis factor-α (TNF-α), IL-2, IL-6, etc., to induce pyroptosis of tumor cells and release a large number of damage-associated molecular patterns (DAMPs) (Giavridis et al., 2018; Shimabukuro-Vornhagen et al., 2018). Meanwhile, they can also directly activate the other immune or non-immune cells, such as the monocyte/macrophage system, through inflammatory cytokines, perpetuating the vicious cycle of inflammation (Mosser and Edwards, 2008). In CRS, macrophages are considered the primary source of pro-inflammatory cytokines, such as IL-1, IL-6, IL-10, IFN-γ, granulocyte-macrophage colony-stimulating factor (GM-CSF), and inducible nitric oxide synthase (iNOS), among others (Hao et al., 2020). Additionally, macrophage pyroptosis and further DAMPs leakage amplify the inflammatory cascade, and secreted catecholamines can also promote the release of other cytokines, thus causing a distortion of the cytokine network (Johnson et al., 2005; Flierl et al., 2007; Shaked et al., 2015). These cytokines may lead to endothelial cell activation by regulating the angiopoietin-TIE2 axis and releasing NO, subsequently resulting in hemodynamic instability, capillary leakage, edema, organ hypoperfusion, cardiovascular dysfunction, and consumptive coagulopathy, and promoting cytokine production, thereby exacerbating CRS (Gust et al., 2017; Hay et al., 2017; Chou and Turtle, 2019). In cancer survivors treated with CAR T-cell therapy, persistent endothelial activation leads to endothelial damage, driving chronic inflammation, hypertension, and a prothrombotic state that accelerates the development of atherosclerosis (Choi et al., 2025).

Among the cytokines involved, IL-6 is the most crucial cytokine mediating CRS toxicity (Lee et al., 2014). Its level is positively correlated with the severity of CRS, and it is a major driver of the inflammatory activation cascade (Shimabukuro-Vornhagen et al., 2018; Chen et al., 2022). IL-6 mainly interacts with another membrane protein, gp130, either through “cis-signaling” of the membrane-bound IL-6 receptor (mIL-6R) or through “trans-signaling” of the soluble IL-6 receptor (sIL-6R), activating the janus kinase/signal transducer and activator of transcription 3 (JAK/STAT3) signaling pathway (Neelapu et al., 2018). This leads to abnormal myocardial electrical activity and a tendency towards arrhythmia, thus affecting normal cardiac electrical activity (Alí et al., 2018). It has been shown that IL-6 gene knockout significantly reduced cardiomyocyte apoptosis in a mouse model of dilated cardiomyopathy (Li Q. et al., 2019). In addition, in sepsis-induced myocardial dysfunction model, IL-6/STAT3 pathway interferes with endoplasmic reticulum (ER) and mitochondrial function by regulating the expression of proteins located in mitochondria-associated ER membranes, increasing intracellular calcium level and leading to myocardial apoptosis (Jiang et al., 2021). At the same time, IL-6/STAT3 pathway may also activate PERK pathway, leading to imbalance of intracellular calcium homeostasis and aggravation of myocardial dysfunction (Men et al., 2023). Blockade of the IL-6 signaling pathway results in the downregulation of pro-inflammatory cytokine secretion and the resolution of most clinical manifestations of CRS (Li Y. et al., 2019; Sterner et al., 2019). TNF-α can also induce the expression of adhesion molecules in vascular endothelial cells by activating the NF-κB signaling pathway, increase ROS production, and promote inflammatory cell infiltration, leading to endothelial injury (Zhou et al., 2017). In sepsis, IL-1β acts synergistically with TNF-α to aggravate myocardial injury by disrupting the blood-brain barrier and increasing mitochondrial membrane permeability, mitochondrial dysfunction and energy metabolism disorders, which aggravate inflammation and oxidative stress (Liu et al., 2025).

## Pre-treatment assessment and post-infusion monitoring for cardiotoxicity

In real-world practice, factors such as advanced age, high tumor burden, and baseline heart conditions may increase the risk of cardiotoxicity due to reduced cardiac functional reserve or an additive effect of CAR T toxicity on pre-damaged myocardium, but these patients still routinely receive CAR T-cell therapy (Costanzo et al., 2024). For example, one study found that patients with underlying cardiac conditions had a higher rate of cardiovascular events after CAR T-cell therapy that correlated with the severity of CRS (Lefebvre et al., 2020). Therefore, pre-treatment assessment of cardiovascular health and risk is crucial for patients who require more intensive monitoring. The clinical practice guidelines issued by the American Society of Clinical Oncology (ASCO) outline evidence-based recommendations for the prevention and monitoring of cancer treatment-related cardiotoxicity (Slivnick et al., 2020). These recommendations commence with a detailed history of cardiovascular symptoms and physical examination, especially screening for cardiovascular risk factors prior to the initiation of treatment (Armenian et al., 2017). All patients scheduled to receive CAR T-cell therapy should undergo a comprehensive baseline cardiac screening using cardiac biomarkers (cardiac troponin and natriuretic peptides), 12-lead electrocardiogram (ECG), transthoracic echocardiogram, cardiac magnetic resonance imaging (MRI), stress tests, etc. (Dalal et al., 2022; Ganatra et al., 2022; Lyon et al., 2022; Camilli et al., 2023a). Collectively, these may provide additional key insights into the ability to tolerate hemodynamic changes during treatment.

After treatment infusion, it is recommended to repeat cardiac monitoring at 7 days and 3 months post-treatment, including assessment of cardiac biomarker levels, 12-lead ECG, and echocardiogram (Cho et al., 2024). Higher peak levels of inflammatory factors such as IL-6 and IL-1, C-reactive protein, ferritin, and troponin, together with CRS, may serve as prognostic markers of cardiotoxicity in patients receiving CAR T-cell therapy (Mahmood et al., 2023). Patients can be divided into low-risk, intermediate-risk, and high-risk groups based on their baseline N-terminal pro brain natriuretic peptide (NT-proBNP) and troponin levels. In high-risk patients, weekly echocardiography is recommended for 1 month and then every 3 months for 1 year. Patients at intermediate risk can be checked every 3 months; Low-risk patients can be tested every 6 months. At the same time, cardiac oncology clinics can consider equipping patients with wearable ECG monitors to detect arrhythmia in time and capture paroxysmal atrial fibrillation or premature ventricular contractions during CRS recovery, so as to take targeted interventions, but manual review is required simultaneously (Cardiovascular Expert Committee of Chinese Medical Doctor Association of Laboratory Medicine, 2024). Patients in whom CRS of grade ≥2 is detected, as well as those with new-onset or deteriorating heart failure or arrhythmia, should be hospitalized for cardiac MRI or multiple-gated acquisition (MUGA) scan, and the case should be reviewed by the cardio-oncology team (Avelar et al., 2017; Jeong et al., 2019). Subclinical fibrosis, as detected by cardiac MRI with measures such as late gadolinium enhancement and extracellular volume fraction, can be a useful tool for predicting late-onset arrhythmias. Studies have shown that the degree of myocardial fibrosis correlates with arrhythmic burden and that fibrosis predicts arrhythmic events even in patients with higher LVEF (Gulati et al., 2013; Bourke et al., 2024). Additionally, the ASCO guidelines recommend that for asymptomatic high-risk patients, echocardiography monitoring should be repeated 6–12 months after the completion of treatment (Armenian et al., 2017).

## Therapeutic management measures for cardiotoxicity

Given the close association between CRS and CAR T cell related cardiotoxicity, mitigating the progression of CRS to more severe grades is of utmost importance in reducing the risk of cardiotoxicity (Alvi et al., 2019; Lefebvre et al., 2020). The grading of CRS is helpful in guiding treatment. Supportive care and antipyretics are commonly used to manage mild CRS, including inotropic support with dobutamine or milrinone when necessary (Faqihi et al., 2020; Ashkar et al., 2025). The IL-6 receptor antagonist tocilizumab was approved by the FDA for the treatment of CRS in 2017 and is currently considered as the first-line treatment for severe CRS secondary to CAR T-cell therapy (Le et al., 2018). Corticosteroids such as dexamethasone and methylprednisolone are regarded as the second line treatment for CRS refractory to anti-IL-6R therapy (Lakomy et al., 2023; Mulvey et al., 2025). Ongoing clinical trials of the IL-1 antagonist anakinra aim to demonstrate its role as an emergency treatment option (Le et al., 2018; Norelli et al., 2018; Camilli et al., 2023b). Typically, CRS requires treatment in an intensive care unit (ICU) for the administration of vasoactive and ionotropic medications, along with aggressive respiratory support (Bellal et al., 2024). In addition, strategies such as fractionated dosing, optimization of the CAR structure, drugs targeting the JAK signaling pathway (such as ruxolitinib and itacitinib), tyrosine kinase inhibitors (such as dasatinib and ibrutinib), and other rational therapies (such as the use of catecholamines or atrial natriuretic peptide, blocking TNF-α, etc.) can also be used for management (Figure 1) (Parampalli Yajnanarayana et al., 2015; Mestermann et al., 2019; Huarte et al., 2020; Huarte et al., 2021). When formulating management strategies, factors such as the pre-treatment tumor burden and tumor type should be taken into account. This is to alleviate symptoms without compromising the therapeutic benefits of this potentially curative approach (Cobb and Lee, 2021).

## Conclusion and perspectives

In summary, with the expanded use of CAR T-cell therapy, the survival rate of cancer patients has increased. However, the treatment is limited by life-threatening CRS and associated cardiotoxicity. Understanding the mechanisms underlying cardiac injury and managing the cardiovascular toxicity of treatment approaches will become increasingly important, as this will enable monitoring, early intervention, and potential prevention of cardiotoxicity (Simela et al., 2025). Novel cardiac biomarkers and specific monitoring parameters not only help to detect the early signs of toxicity and its severity, but also contribute to enhancing clinical management and providing personalized medical care to patients (Christenson et al., 2015; Singh et al., 2015; Henri et al., 2016). Incorporating cardiovascular toxicity monitoring into clinical trials will play a crucial role in improving the safety of CAR T-cell therapy for cancer patients and fully realizing its therapeutic potential (Cho et al., 2024). However, the assessment of cardiotoxic outcomes may be biased by the selection of patients in early-phase clinical trials and selective reporting in retrospective studies. In recent years, various authorities are trying to establish the standardization of cardiotoxicity monitoring of CAR T-cell therapy, for example, the European Society of Cardiology (ESC), American Heart Association (AHA) and so on. Better preventive and treatment methods can be developed through prospective studies of patients and multidisciplinary integration among oncologists, oncohematologists, cardiologists, and other specialists, so as to promote wider application of this new therapy (Zhao et al., 2024).
